# Development and Validation of the Tibetan Primary Care Assessment Tool

**DOI:** 10.1155/2014/308739

**Published:** 2014-05-21

**Authors:** Wenhua Wang, Leiyu Shi, Aitian Yin, Youwen Lai, Elizabeth Maitland, Stephen Nicholas

**Affiliations:** ^1^Center for Health Management and Policy, Shandong University, 44 Wenhuaxilu, Jinan, Shandong 250012, China; ^2^Johns Hopkins Bloomberg School of Public Health, Johns Hopkins Primary Care Policy Center, 624 North Broadway, Baltimore, MD 21205, USA; ^3^Tibet Health Capacity Building Program, No. 2 Norbulinka South Road, Lhasa, Tibet 850000, China; ^4^School of Management, Australian School of Business, University of New South Wales, Sydney, NSW 2052, Australia; ^5^Guangdong Research Institute for International Strategies, Guangdong University of Foreign Studies, No. 2 Baiyun Dadao Bei, Guangzhou, Guangdong 510420, China; ^6^Tianjin Normal University, 57-1 Wujiayao Road, Hexi District, Tianjin 300074, China; ^7^Faculty of Business and Law, University of Newcastle, Newcastle, NSW 2308, Australia

## Abstract

*Objective*. To develop a primary care assessment tool in Tibetan area and assess the primary care quality among different healthcare settings. *Methods*. Primary care assessment tool-Tibetan version (PCAT-T) was developed to measure seven primary care domains. Data from a cross-sectional survey of 1386 patients was used to conduct validity and reliability analysis of PCAT-T. Analysis of variance was used to conduct comparison of primary care quality among different healthcare settings. *Results*. A 28-item PCAT-T was constructed which included seven multi-item scales and two single-item scales. All of multi-item scales achieved good internal consistency and item-total correlations. Scaling assumptions tests were well satisfied. The full range of possible scores was observed for all scales, except first contact and continuity. Compared with prefecture hospital (77.42) and county hospital (82.01), township health center achieved highest primary care quality total score (86.64). *Conclusions*. PCAT-T is a valid and reliable tool to measure patients' experience of primary care in the Tibet Autonomous Region. Township health center has the best primary care performance compared with other healthcare settings, and township health center should play a key role in providing primary care in Tibet.

## 1. Introduction

Mounting evidence has demonstrated that primary care may contribute to better health outcomes and lower health care costs [1–9]. The US Institute of Medicine defined primary care as “the provision of integrated, accessible health care services by clinicians who are accountable for addressing a large majority of personal health care needs, developing a sustained partnership with patients, and practicing in the context of family and community” [10, 11]. In order to assess and monitor primary care performance, there is a need to develop valid and reliable instruments. Highlighting the fallibility of relying on unidimensional proxies for primary care, Safran et al. showed the value of measuring primary care in multidimensional terms that parallel its formal definition [12]. Under this framework, several multiscale instruments were developed, including the primary care assessment tool (PCAT) [12, 13]. PCAT was developed by Johns Hopkins Primary Care Policy Center and included four unique domains (first contact, longitudinality, comprehensiveness, and coordination) as well as three derivative attributes (family centeredness, community orientation, and cultural competence) [13]. Its original English version has been translated into Spanish, Portuguese, Chinese (PCAT-C), and Korean versions, and testing has confirmed that all of these versions have good validity and reliability in terms of congruence between the theoretically derived measures and the empiric results in terms of the underlying structure of the principal primary care domains [14–18].

During the past four years of national health reform, the Chinese central government and the Tibet Autonomous Region (TAR) government have made significant investments in the Tibetan health system, especially in the primary care system. In the context of rapidly changing health care investments, there is a particular need to measure primary care performance in the TAR. PCAT-C provides a starting point for assessing primary care in the TAR. The original English PCAT version, with more than 90 items, was found unsuitable for China's health system, resulting in a PCAT-C modified to fit the local Chinese context [17]. Unfortunately, we cannot use PCAT-C directly in TAR. First, most residents cannot speak Chinese. Second, there are substantial differences in Chinese versus Tibetan health care providers, especially with respect to geographic accessibility. TAR is located in southwestern China, with an average elevation of 4900 meters and an area about one-eighth of China's area. The geography of this large and inaccessible province contrasts with its population of roughly three million, which, although overwhelmingly  Tibetan, also comprises Monpas, Qiang, and Lhobas indigenous populations, as well as Han and Hui people. Therefore, a PCAT-Tibetan (PCAT-T) is required.

TAR has seven prefectures, 74 counties, 691 towns, and 5254 villages. In each prefecture, there are two types of prefecture level hospitals: prefecture people's hospitals, which provide western medicine service, and prefecture Tibetan medicine hospitals, which provide traditional Tibetan medicine services. In this paper, we do not differentiate between the two types of prefecture hospitals. Every county has one county hospital, every town has one township health center, and every administrative village has one village clinic. Except for village clinics, all of these organizations are main primary care providers. The low medical capacity of village clinics is reflected in their doctors' rudimentary three months' training in a prefecture health school, a fixed monthly salary of only 600 RMB, and the lack of adequate village medical center sites, with many village doctors practicing from their own houses. Seeking jobs outside the health system, many village physicians practiced medicine part-time. For these reasons, we excluded village clinics from the study.

While the primary care system in TAR shares similarities with inland China, there is the need both to translate the PCAT-C into Tibetan and to assess differences between the TAR and inland Chinese health care systems. In addition to setting out the modified PCAT-T version of the PCAT-C instrument, our study conducts a psychometric evaluation of the PCAT-T version, based on a sample of 1440 interviews across 11 health care sites, ranging from prefecture level hospitals to township health centers. The paper then compares primary care achievement across these different health care settings, encompassing sites in two of the seven TAR prefectures.

## 2. Materials and Methods

### 2.1. Measurement

The adaption of PCAT-C for the TAR was carried out in the following steps. First, the PCAT-C was translated into Tibetan by two health staff, who were fluent in both Tibetan and Chinese. Second, seven health experts reviewed the translated PCAT-T version. The expert panel included two health service researchers, who are familiar with Tibet's primary care system, one county health bureau director, one prefecture health bureau director, and three doctors from local TAR hospitals. Experts rated the necessity of each item using a five-point scale (1 = “irrelevant” to 5 = “100% relevant”). They were also asked to add items they believed were necessary based on the Tibetan local environment. Consensus was reached regarding whether to alter, remove, or add an item. Third, pilot testing was conducted through qualitative interviews with 20 random patients for further modification. Finally, through in-depth discussions with two health services researchers, the final PCAT-T was constructed.

The expert review scored highly (more than 4 in a scale of 1 “not relevant” to 5 “highly relevant” to Tibet) the 36-item PCAT-C as a measure of patients' experience of primary health care quality covering the six domains of first contact, continuity, coordination, comprehensiveness, family-centeredness, and community orientation. Since the PCAT-C items were deemed appropriate for the Tibetan context, all the PCAT-C items were retained in the PCAT-T version.

The PCAT-C version reduced both the items and the principal primary care domains compared with the original English version (see Appendix 1 of Yang et al. [17]). The expert panel recommended one item from the English version to be reinstated in the PCAT-T version to measure “cultural competence” in the patients' experience of primary care quality. Culturally competent care refers to care that honors and respects the beliefs, interpersonal styles, attitudes, and behaviors of people receiving health care. The item to measure cultural competence sought information on whether a patient would recommend their primary care provider to a friend or relative. In common with the original English PCAT version, but not the PCAT-C, this item in the PCAT-T reflects the diverse cultures and languages in TAR.

Further, the expert review recommended a number of additional questions, including a measure of the regular source of care. In the original English version, there are three questions to measure regular source of care and two questions in PCAT-C. Based on the results of group discussion, the expert panel took the view that two or three questions were difficult for local patients to understand and so recommended just one question to measure regular source of care. Four additional questions were used to measure patients' satisfaction of their regular source of care. A four-point Likert-type scale was applied to measure certainty as to whether a service was received, ranging from “1” (“definitely not”) to “4” (“definitely”). A neutral response of “not sure/don't remember” was provided for the lack of knowledge about a characteristic.

### 2.2. Data Collection

A stratified, purposive sampling approach was used to select study sites. Socioeconomic and geographic factors were employed to ensure our selection of sampling sites represented Tibetan health agencies at each level. Given the socioeconomic level and geographic location of TAR's seven prefectures, two prefectures were sampled: Shigatse, located in western Tibet with 18 low socioeconomic level counties, and Linzhi in eastern Tibet with seven high socioeconomic level counties.

In Shigatse prefecture, we selected two prefecture level hospitals; at the county level, the Jiangzi and Lazi hospitals; and two health centers per sampling county at the township level. In Linzhi prefecture, we selected two prefecture level hospitals; the Gongbujiangda county hospital; and two township health centers in Gongbujiangda county. The sample sizes were comparable to three key studies that showed 300 interviews were required at each sample site for comparison analysis [19–21]. Considering some collected questionnaires may contain missing data, 10 additional questionnaires were conducted at each township health centre, 20 additional questionnaires at each county hospital, and 30 additional questionnaires at each prefecture level hospital. Overall, four prefecture level hospitals (720), three county hospitals (360 interviews), and six township health centers (360 interviews) yielded 1440 interviews.

The data were collected between September and October 2013 by trained interviewers from the local health bureau through face-to-face interviews with patients 18 years old or older, who had completed their visits to township health centers or hospital outpatients. Patients were given small gifts (worth 10 RMB) of appreciation upon completion of the interview. While 1440 questionnaires were administered, 54 questionnaires were deleted due to missing data, leaving 1386 completed questionnaires.

### 2.3. Analysis

For consistency with methods used in PCAT studies in other countries, we assigned a median value of 2.5 to “not sure/don't remember” answers, to be consistent with the methods used in PCAT studies in other countries [17]. We imputed missing values using multiple regression based on the assumption that missing values are random. We converted Likert scales to scores ranging from 25 to 100 by dividing the Likert scale by 4 and multiplying by 100. Then, we conducted the validation of PCAT-T in the following steps. First, we used factor analysis (principal component analysis and varimax rotation) to measure construct validity. To attain the best fitting structure and the correct number of factors, the following criteria were used: eigenvalues > 1.0 and factor loadings > 0.35. Before conducting the factor analysis of the PCAT-T, the Kaiser-Meyer-Olkin and Bartlett's test was calculated to evaluate whether the sample was large enough to perform a satisfactory factor analysis.

Second, the data's internal consistency reliability was assessed by Cronbach alphas and item-total correlation. For a scale to be considered sufficiently reliable, an alpha value of 0.7 is recommended. All the retained items should exceed the minimum acceptable item-total correlation of 0.30.

Next, item-convergent validity and item-discriminant validity were tested by item-scale correlations and scaling success rate. The range of item-scale correlations was used to test equal item-scale correlation, while intraclass correlation was used to measure equal item variance. Then, descriptive statistics were performed for the revised primary care scales, including mean, standard deviation, median, and interscale correlation. Finally, primary care achievement of different health care settings was compared using analysis of variance [12, 18, 22].

## 3. Results

As reported above, a total of 1386 completed interviews were used.  Table 1 shows patients' sociodemographic (age, gender, education, occupation, income, and marital status), health care utilization (PCP visits and inpatient experience), and self-assessed health details. Among 1386 participants, 46.4% were male; the average age was 41.72 years old; 76.9% were married; about one-third received junior high school education or above; and 80.4% were employed. For nearly half the sample, the annual household income was above 30000 RMB. One-fifth of the sample received inpatient care during the past year, and more than 60% reported good, self-rated health. TAR's recent census does not include information on the sociodemographics of Tibetan population over 18 years old. However, the average family income in the census and our sample (RMB 30000) and the gender breakdown (51% versus 46% male) were roughly comparable. Our sample had a higher education level than the Tibetan population because we sampled only patients over 18 years old (who had the opportunity to complete a junior high school education).

### 3.1. Construct Validity

The calculated Kaiser-Meyer-Olkin and Bartlett's test statistic was 0.923 with a *P* value < 0.001, indicating that the sample was large enough to perform a satisfactory factor analysis. All 37 items were included in the principal component analysis and nine components were derived, based on the criteria that the eigenvalues were larger than 1.0. Nine items from the original scale were eliminated, because their secondary loadings were > 0.35: two items from first contact-accessibility, three items from original continuity, one item from original comprehensiveness, two items from original coordination, and one item from family centeredness. Factor loadings of all the retained items ranged from 0.40 to 0.81, above the standard of 0.35.

Finally, a 28-item instrument was constructed which included seven multi-item scales and two single-item scales. The seven multi-item scales were first contact and continuity (6 items); comprehensiveness (medical care) (4 items); comprehensiveness (social care) (3 items); first contact (access) (2 items); coordination (2 items); family centeredness (5 items); and community orientation (4 items). Two single-item scales were same doctor and stableness (see Table 2).

### 3.2. Internal Consistency

The overall Cronbach alpha coefficient of PCAT-T was 0.92. Cronbach alpha coefficient results were above 0.7 for all multi-item scales, except first contact (access) scale (0.63). As presented in Table 2, the corrected item-total correlations ranged from 0.42 to 0.74, far above the standard of 0.30.

### 3.3. Scaling Assumption Testing

All item-scale correlations exceeded 0.6, with the majority above 0.7, except two items in the first contact and continuity scale (0.61, 0.67) and one item in the family centeredness scale (0.68). All items had higher correlation with their own scale than with other scales, achieving 100% scaling success. As shown in Table 3, all scales demonstrated a relatively narrow range of item-scale correlations (from 0.01 for “first contact (access)” to 0.15 for “comprehensiveness (medical care)”).

### 3.4. Descriptive Features of the PCAT-T

We identified seven multi-item scales and two single-item scales, which explained 60.7% of the common variance in the responses to 28 of the original 37 items in the PCAT-T. The alpha coefficient of each scale substantially exceeded its correlation with all other primary care scales.  Table 4 presents estimates of central tendency and dispersion of score distribution for seven multi-item scales and two single-item scales. The full range of possible scores was observed for all scales, except first contact and continuity.

### 3.5. Primary Care Quality in Various Settings

Table 5 shows that township health centers achieved the highest total primary care quality score (86.64), followed by district hospitals (82.01), while prefecture hospitals achieved the lowest scores (77.42). For each scale, township health centers also achieved the highest scores, with the exception of the same doctor and stableness scales.

## 4. Discussion

The PCAT-T is not a simple translation of the PCAT-C into Tibetan. The expert review identified key modifications to the PCAT-C version to reflect the Tibetan context. A standard psychometric evaluation method was then used to evaluate the PCAT-T version. Overall, the PCAT-T achieved good validity and reliability. The final PCAT-T consisted of seven multi-item scales and two single-item scales. Although the final PCAT-T scales were not completely consistent with the PCAT theoretical domains, the final nine scales covered seven domains suggested by PCAT. Three scales (first contact, continuity, and coordination) in PCAT were split into five scales (first contact and continuity, first contact (access), coordination, the same doctor, and stableness) in the PCAT-T and one scale (comprehensiveness) in PCAT was represented by two scales (comprehensiveness (medical care) and comprehensiveness (social care)) in the PCAT-T. Family centeredness and cultural competency in PCAT were integrated into family centeredness in the PCAT-T. There was no difference in the community orientation scale between PACT-C and PACT-T. All seven multi-item scales achieved relatively good internal consistency. Therefore, PCAT-T is a valid and reliable tool to measure patients' experience of primary care in the TAR.

In the TAR, township health centers, county hospitals, and prefecture hospitals are the main primary care providers. From the PCAT-T results among different healthcare settings, we found that township health centers achieved the highest score, especially on the scale of first contact (access), which means patients can receive health care without waiting for a long time (less than thirty minutes) and can receive the needed service more easily in township health centers. As residents in the TAR are distributed across a dispersed area, geographic accessibility of health care is a significant problem. To address this problem, the TAR has invested in capacity building for township health centers to provide better primary care, because residents in a town are concentrated and the health staff in township health centers are more familiar with the covered residents. For township health centers, the national government funds staff salaries, infrastructure, and equipment cost, supporting, on average, 4.6 health staff and providing 7-day 24-hour health services. Our results provide evidence that TAR's investment in township health centers achieved a better outcome than county hospitals (with their larger geographical cover) and prefecture-level hospitals (with the most difficult geographic accessibility and less familiar doctor-patient relationships).

However, township health centers received the lowest score on the scale of the same doctor. To promote capacity building, TAR's regional health policy allocated new medical graduates to township health centers, but these doctors quickly transferred to upper level health facilities as opportunities arose. This led to township health centers' low score on the scale of same doctor.

Our study has several limitations. First, patient-reported measurement is subject to recall bias. Some aspects of technical quality cannot be assessed from patients' perceptions, because of their limited clinical knowledge. Despite these issues, patient reporting is widely accepted as a method of measuring aspects of care important to patients [23]. Second, we assumed that each respondent had experiences of visiting other health care providers in addition to their regular source of care, because this was a precondition to measure achievement of coordination scale. Based on our study experience, most respondents had such experience. Even if respondents did not have such experience, we argue they could make a judgment about items in the coordination scale on the basis of their past experiences in their regular source of care. Third, we only used one question (what is the place you usually go to when getting sick or seeking an advice for health?), instead of the original three questions to measure patients' regular source of care. Given the challenges stemming from geographic accessibility, patients generally have no choice but to attend the site that is the closest in distance to their place of residence. Fourth, three scales (first contact (access), the same doctor, and stableness) have lower correlations with other scales. Most of these correlation coefficients are below 0.2 and the alpha coefficient of first contact (access) scale is below 0.7, which suggest further improvement of PCAT-T, especially in the scale of first contact (access).

## 5. Conclusion

PCAT-T is a valid and reliable tool to measure patients' experience of primary care in the Tibet Autonomous Region. Township health center has the best primary care performance compared with other healthcare settings, and township health center should play a key role in providing primary care in Tibet. Using the PCAT-T, future work should be conducted to analyse two key aspects of TAR health care reform and performance. The first is to explore characteristics from the provider level and organization level that lead to different primary care performance. The other is to examine the extent to which the principal scales of primary care can be linked to health outcomes.

## Figures and Tables

**Table 1:** Descriptive statistics for study sample (*n* = 1386).

Characteristics	Respondents	*n*	%
Healthcare setting	Prefecture hospital	692	49.9
	County hospital	336	24.2
	Township health center	358	25.8
*Sociodemographic characteristic *			
Gender	Male	643	46.4
	Female	743	53.6
Age	<60 years	1224	88.3
	≥60 years	162	11.7
Education	Below junior high school	931	67.2
	Junior high school and above	455	32.8
Occupation	Employed	1114	80.4
	Unemployed	272	19.6
Annual household income (RMB)	≤30000	783	56.5
	>30000	603	43.5
Marital status	Unmarried	320	23.1
	Married	1066	76.9
*Health service utilization *			
Number of PCP visits during the past year	0-1	376	28.4
	2-3	546	44.1
	≥4	464	27.5
Whether inpatient during the past year	Yes	291	21.0
	No	1095	79.0
*Health characteristics *			
Self-rated health	Poor health	511	36.9
	Good health	875	63.1

**Table 2:** Results of principal component analysis and internal consistency analysis.

Scale	Number of items	Item loadings on the component	Item-total correlations	Alpha	Variance (%)
First contact and continuity	6	0.40–0.73	0.44–0.58	0.78	9.2
Comprehensiveness (medical care)	4	0.56–0.80	0.49–0.69	0.78	7.8
Comprehensiveness (social care)	3	0.43–0.81	0.42–0.59	0.70	5.3
First contact (access)	2	0.80	0.46	0.63	4.5
Coordination	2	0.47–0.66	0.55	0.71	6.8
Family centeredness	5	0.49–0.69	0.48–0.60	0.77	9.9
Community orientation	4	0.72–0.79	0.64–0.74	0.85	10.3
Same doctor	1	0.52	—	—	3.6
Stableness	1	0.77	—	—	3.3

Total	28	—	—	0.92	60.7

**Table 3:** Results of scale assumptions analysis.

Scale/item	Number of items	Item-scale correlations	Item, other scale correlations	Scaling success rate (%)	Intraclass correlations
First contact and continuity	6	0.61–0.72	0.01–0.48	100	0.35
Comprehensiveness (medical care)	4	0.70–0.85	0.05–0.40	100	0.45
Comprehensiveness (social care)	3	0.73–0.84	0.09–0.45	100	0.43
First contact (access)	2	0.85-0.86	0.01–0.27	100	0.43
Coordination	2	0.87–0.89	0.01–0.51	100	0.55
Family centeredness	5	0.68–0.76	0.02–0.47	100	0.39
Community Orientation	4	0.80–0.86	0.01–0.47	100	0.59
Same doctor	1	—	—	—	—
Stableness	1	—	—	—	—

**Table 4:** Descriptive features of PCAT-T^a^.

Scale	Number of items	Mean	Standard deviation	Median	Score range
First contact and continuity	6	89.24	12.00	91.67	33–100
Comprehensiveness (medical care)	4	79.99	18.25	81.25	25–100
Comprehensiveness (social care)	3	84.93	15.74	85.63	25–100
First contact (access)	2	67.46	24.58	62.50	25–100
Coordination	2	82.90	18.49	87.50	25–100
Family centeredness	5	86.68	13.30	90.00	25–100
Community orientation	4	75.84	20.40	79.69	25–100
Same doctor	1	71.52	25.86	75.00	25–100
Stableness	1	46.52	22.67	50.00	25–100

Total	28	80.92	10.28	82.14	42–100

^a^Primary care assessment tool-Tibetan version.

**Table 5:** Primary care quality of different settings in Tibet.

Scale	Township health center score mean (SE)	County hospital score mean (SE)	Prefecture hospital score mean (SE)	*P* value
First contact and continuity	95.40 (0.39)	90.93 (0.52)	85.23 (0.51)	<0.001
Comprehensiveness (medical care)	80.9 (0.99)	78.05 (1.03)	80.45 (0.67)	0.075
Comprehensiveness (social care)	90.33 (0.84)	85.39 (0.73)	81.91 (0.61)	<0.001
First contact (access)	80.16 (1.15)	69.21 (1.40)	60.04 (0.86)	<0.001
Coordination	91.65 (0.73)	84.39 (0.91)	77.65 (0.75)	<0.001
Family centeredness	90.34 (0.68)	87.93 (0.60)	84.18 (0.53)	<0.001
Community orientation	87.98 (0.87)	80.36 (0.85)	67.36 (0.78)	<0.001
Same doctor	66.42 (1.54)	70.95 (1.26)	74.44 (0.95)	<0.001
Stableness	45.37 (1.18)	43.14 (1.04)	48.75 (0.92)	0.001

Total	86.64 (0.49)	82.01 (0.50)	77.42 (0.38)	<0.001

## Abbreviations

PCAT: Primary care assessment tool
PCAT-C: Primary care assessment tool-Chinese version
PCAT-T: Primary care assessment tool-Tibetan version.
